# Insured Patients

**Published:** 1914-01-10

**Authors:** H. M. Howard

**Affiliations:** Hon. Secretary Lynn and West Norfolk Hospital. Lynn and West Norfolk Hospital, King's Lynn


### THE EDITOR'S LETTER-BOX.

### Insured Patients.

*To the Editor of* The Hospital.

Sir,—In view of the importance to all hospital authorities of the National Insurance Act the following particulars, prepared for the Board of Management of the Lynn and West Norfolk Hospital, may be of some general interest. During a period of fifteen weeks, terminated November 25 last, the patients admitted to this institution were classified as "Insured" or otherwise, and the following proportion was shown :—

Of 194 in-patients : 49, or 25 per cent., were insured patients; 16, or 8 per cent., had been sent by school authorities; 129, or 67 per cent., were other patients. Of 250 out-patients : 24, or 9.6 per cent., were insured persons, and the remainder otherwise. Of 255 cases in the casualty department : 56, or 22 per cent., were insured persons, and the remainder otherwise.

It will appear from the above that in the future 25 per cent. of the beds of this hospital will be needed for the accommodation of insured persons. The only direct subscription received up to the present for this work has been a contribution from two patients who had no dependants. Some particulars of the experience of similar institutions would, I believe, be of great value.—I am, Sir, yours faithfully,

H. M. Howard, Hon. Secretary Lynn and West Norfolk Hospital.

Lynn and West Norfolk Hospital,
King's Lynn, January 5, 1914.

[We shall be grateful if other hospital Secretaries will forward to us information of a similar kind.—Ed.]

